# Quantification and isolation of *Bacillus subtilis* spores using cell sorting and automated gating

**DOI:** 10.1371/journal.pone.0219892

**Published:** 2019-07-29

**Authors:** Marianna Karava, Felix Bracharz, Johannes Kabisch

**Affiliations:** Computer-aided Synthetic Biology, Institute for Biology, Technische Universität Darmstadt, Darmstadt, Germany; Università degli Studi di Pavia, ITALY

## Abstract

The Gram-positive bacterium *Bacillus subtilis* is able to form endospores which have a variety of biotechnological applications. Due to this ability, *B*. *subtilis* is as well a model organism for cellular differentiation processes. Sporulating cultures of *B*. *subtilis* form sub-populations which include vegetative cells, sporulating cells and spores. In order to readily and rapidly quantify spore formation we employed flow cytometric and fluorescence activated cell sorting techniques in combination with nucleic acid fluorescent staining in order to investigate the distribution of sporulating cultures on a single cell level. Automated gating procedures using Gaussian mixture modeling (GMM) were employed to avoid subjective gating and allow for the simultaneous measurement of controls. We utilized the presented method for monitoring sporulation over time in germination deficient strains harboring different genome modifications. A decrease in the sporulation efficiency of strain Bs02018, utilized for the display of sfGFP on the spores surface was observed. On the contrary, a double knock-out mutant of the phosphatase gene encoding Spo0E and of the spore killing factor SkfA (Bs02025) exhibited the highest sporulation efficiency, as within 24 h of cultivation in sporulation medium, cultures of BS02025 already consisted of 80% spores as opposed to 18% for the control strain. We confirmed the identity of the different subpopulations formed during sporulation by employing sorting and microscopy.

## Introduction

*Bacillus subtilis* is a model organism extensively studied for its ability to differentiate depending on the growth conditions [1]. Sporulation in *B*. *subtilis* is one of the most thoroughly investigated cellular differentiation programs, triggered by a combination of signals including nutrient exhaustion and cell density [2,3]. The starving cells undergo an asymmetric division, governed by a complex regulatory network which results in the formation of metabolically inactive spores [4,5]. The levels of the master regulator of this process, Spo0A in its phosphorylated form define whether a cell will enter the sporulation pathway [6]. However, even under optimal sporulating conditions, only a part of the population undergoes sporulation [7,8]. Several studies report on the heterogeneity of sporulation as the outcome of microenvironmental signals in combination with genetic fluctuations and stochasticity [6,7,9].

*B*. *subtilis* endospores have been investigated for many years in an effort to elucidate their genetic regulation, their biochemical properties and their morphology [4,10,11]. A set of unique properties such as resilience to environmental assaults, in combination with the potential of the spore coat to be utilized as a scaffold for protein display, laid the ground for numerous biotechnological applications. To date, spores have been utilized as platforms for display of a range of biotechnologically interesting peptides, including enzymes and antigens [12,13].

Throughout the years, methods have been developed for studying different aspects of sporulation in various species including microscopy, microfluidic chips in combination with fluorescence imaging, Raman spectroscopy and Fourier-transform infrared spectroscopy (FTIR) [14–17]. However many described techniques either require special instrumentation or are laborious and not automatable. One of the oldest techniques to date for spores quantification in culture is via hemocytometers [18]. Despite the low cost, this technique is tedious and not recommended for studies focused on the dynamics of sporulation. Most commonly quality and quantity of sporulation are determined by counting cell forming units after heat shock treatment [19]. This method however is an indirect measure since it can only quantify the survival of the heat treatment. Hence, it can be applied only in cases where the spores are still able to germinate. In contrast, flow cytometry is a useful, rapid and high throughput technique, employed for evaluation of the intrinsic characteristics of cells or particles based on their light scattering properties [20]. Flow cytometry has been employed for many years as a method for single cell analysis and separation of eukaryotic cells. However, so far it has remained challenging to distinguish among bacterial species or small size particles [20,21]. Hence, studies reporting on flow cytometry as a tool for discriminating vegetative cells from spores of *B*. *subtilis* remain rare.

The here presented method uses DNA staining and flow cytometry with automated gating procedures and enables discrimination of three subpopulations formed during sporulation. Initially three different fluorescent dyes were tested in distinct samples of cells and spores and were evaluated for their effectiveness to separate the two populations. After optimizing the staining procedure by employing artificial mixtures of cells and spores, we tested the application of the method in liquid sporulating cultures. We validated the identity of the observed populations by sorting and microscopy. Finally we applied the developed method for monitoring sporulation dynamics of four germination deficient strains carrying different genomic modifications. Our methodology successfully enabled the qualitative and quantitative assessment of sporulation efficiency of the tested strains.

## Materials and methods

### Strain development

Seven different strains of *B*. *subtilis KO7 (Bacillus Genetic Stock Center ID 1A1133*, *derivative strain of B*. *subtilis PY79)* were utilized as test organisms in the present study. All strains used in the present work, are listed in Table 1. Strain Bs02002 was utilized for testing the viability of spores after staining. Strain Bs02005 employed for the production of non-sporulating cells, harbors deletion of *sigE* [22] and *spoIIGA* [23] genes. Strains Bs02003, Bs02018, Bs02020, Bs02025 used for application of the developed method, harbor deletions of *cwlD* and *sleB* genes, leading to a germination deficient phenotype [24–26]. Strain Bs02003 was further employed for staining optimization and classification of different sub-populations formed during sporulation. Strain Bs02018 utilized for display of superfolder green fluorescent protein (sfGFP) (www.addgene.org, plasmid: PYTK001 [27] on the surface of the spores, was constructed by fusing the sequence of *sfgfp* in frame to the C-terminal coding end of the gene *cotB*. The two genes were bridged by a linker made of 12 amino acids. The fusion gene was integrated into the *pksX* locus via homologous recombination and was expressed under the control of P*cotYZ* promoter (*pksX*::*PcotYZ-cotB-linker-sfgfp)*. The native *cotB* gene was deleted from the genome of strain Bs02018 [28]. Strain Bs02020 harbors additionally deletion of *cotA* [29] gene whereas strain Bs02025 harbors deletion of *skfA* [30] and *spo0E* [31] genes. All strains employed in this work were generated by transformation of *B*. *subtilis* with non-replicative plasmid DNA as described previously [32].

**Table 1:** List of strains used in the present study.

Strain	Genotype	Resistance	Reference
*Bacillus subtilis* KO7	*ΔnprE ΔaprE Δepr Δmpr ΔnprB Δvpr Δbpr*	no	*Bacillus Genetic Stock Center ID 1A1133*
Bs02002	*ΔsacA*::*(Zeo*^*R*^, *P*_*xylA*_*-cre*, *P*_*spac*_*-comS*, *lacl)*	zeo^R^	This work
Bs02003	*ΔsacA*::*(Zeo*^*R*^, *P*_*xylA*_*-cre*, *P*_*spac*_*-comS*, *lacl)*, *ΔcwlD*::*lox72*, *ΔsleB*::*lox72*	zeo^R^	This work
Bs02005	*ΔsacA*::*(Zeo*^*R*^, *P*_*xylA*_*-cre*, *P*_*spac*_*-comS*, *lacl)*, ΔsigE/spoIIGA::lox72	zeo^R^	This work
Bs02018	*ΔsacA*::*(Zeo*^*R*^, *P*_*xylA*_*-cre*, *P*_*spac*_*-comS*, *lacl)*, *ΔcwlD*::*lox72*, *ΔsleB*::*lox72*, *ΔpksX*::*(P*_*cotYZ*_*-cotB-linker-sfGFP)*, *ΔcotB*::*lox72*	zeo^R^	This work
Bs02020	*ΔsacA*::*(Zeo*^*R*^, *P*_*xylA*_*-cre*, *P*_*spac*_*-comS*, *lacl)*, Δ*cwlD*::*lox72*, Δ*sleB*::*lox72* Δ*cotA*::*lox72*	zeo^R^	This work
Bs02025	*ΔsacA*::*(Zeo*^*R*^, *P*_*xylA*_*-cre*, *P*_*spac*_*-comS*, *lacl)*, Δ*cwlD*::*lox72*, Δ*sleB*::*lox72*, Δ*skfA*::*lox72*, Δ*spo0E*::*lox72*	zeo^R^	This work

#### Plasmid construction for *B*. *subtilis* deletion strains

The P*cotYZ* promoter (108 bp) was amplified using *B*. *subtilis KO7* genome as template and oligonucleotides 02124 and 02171 (S1 Table). DNA coding for *cotB* gene (1,140 bp) was amplified using *B*. *subtilis KO7* genomic DNA and oligonucleotides pair 02172 and 02173 (S1 Table) encoding for CotB and a flexible linker (2 repeats of Gly-Gly-Gly-Gly-Gly-Ser) located at the C-terminal end of CotB. The *sfgfp* gene (717 bp) was amplified from the plasmid pYTK001 (www.addgene.org, plasmid: PYTK001) using the oligonucleotides 02066 and 02067 (S1 Table). The plasmid vector containing two homology regions for integration in the *pksX* locus and the spectinomycin resistance cassette, was amplified using plasmid pJK179 as template and oligonucleotides 31055 and 02017 (S1 Table). The PCR products amplified using plasmid template, were digested with DpnI (NEB, Ipswich, MA, USA) in order to reduce plasmid carry over. Plasmid assembly was performed using SLiCE as described by Messerschmidt and coworkers [33] using *E*.*coli* DH10B (NEB, Ipswich, MA, USA) as the shuttle host.

Gene deletions in *B*. *subtilis* were conducted via double crossover. Flanking regions upstream and downstream of the gene of interest were PCR amplified using chromosomal DNA as template and the respective oligonucleotide pair (S1 Table). These PCR products, a spectinomycin resistance cassette [34] and a linearized pJET vector (Thermo Fisher Scientific) were assembled using the SLiCE method [33]. The resulting plasmids were isolated from *E*. *coli*, verified via sequencing and transformed without linearization into *B*. *subtilis* via natural competence as described previously [32]. A list of all plasmids used for deletions can be found in S2 Table whereas corresponding Genbank files are available in a public GitLab repository under https://gitlab.com/sporesort/.

### Media and growth conditions

Vegetative cells were grown in Lysogeny broth (LB) composed of 10 g/L tryptone, 5 g/L yeast extract, 5 g/L NaCl (LB5; Carl Roth, Germany) with agitation (200 rpm) at 37°C. Sporulation was induced by nutrient exhaustion as described by Nicholson and Setlow, 1990 [35] including a few modifications. In brief, *B*. *subtilis* strains were inoculated into 4 mL LB containing the antibiotic zeocin (20 μg/mL). Cell cultures were grown overnight at 37°C with agitation (200 rpm). Cells from the overnight cultures were harvested by centrifugation and inoculated into 500 mL Erlenmeyer flasks containing 200 mL prewarmed Difco sporulation medium (DSM) [8 g/L Nutrient broth No. 4, 1 g/L KCl, 1 mM MgSO_4_, 1 mM Ca(NO_3_)_2_, 10 μM MnCl_2_, 1 μM FeSO_4_ (Sigma-Aldrich, Germany)]. Cells were grown at 37°C with agitation (200 rpm). For spore purification, after 72 h of cultivation the cultures were harvested by centrifugation at 3,400 x g for 15 min. The spores were purified using a Renografin gradient centrifugation as described by Nicholson et al. [35] followed by resuspension in 1 x phosphate buffered saline (PBS). The purified spore suspension contained >90% phase bright spores as confirmed by microscopy.

### Preparation of dyes and staining conditions

Two different fluorescent dyes were utilized for the following experiments: SYBR green I (SYBR1) (10,000 x stock, Invitrogen^TM^) and SYBR Green II (SYBR2) (10,000 x stock, Invitrogen^TM^). SYBR green dyes bind to nucleic acids, whereas SYBR2 has higher sensitivity for RNA [36]. SYBR1 and SYBR2 stocks were diluted 1:100 times (100 x stock) with Type I water respectively. For each dye, aliquots of working solutions were transferred in 1.5 mL opaque tubes; aliquots of working dilutions were kept at 4°C. The dyes were tested in different concentrations as well as in combinations. The final tested concentrations used for analysis of SYBR1 and SYBR2 ranged from 1 x to 4 x of the suppliers suggested concentration. Prior to flow cytometry, 500 μL aliquots of cells and spores at an optical density at 600 nm of 0.5 in 1 x PBS, were prepared. For optimization of the staining method, an artificial mixture of non sporulating cells (Bs02005) and spores (Bs02003) with a final optical density at 600 nm of 0.5 and a final volume of 500 μL was prepared. The artificial mixture contained non-sporulating cells and purified spores in a rough 1 to 1 proportion. After resuspension, the samples were mixed with 5 μL, 10 μL or 20μL of each dye working solution respectively and were incubated in room temperature in the dark for 20 min.

### Sample preparation for monitoring sporulation in liquid cell cultures

For each experiment prior to sporulation, precultures were prepared from stock cultures stored at -20°C by streaking the stock solution onto LB agar plates containing zeocin (20 μg/mL). After overnight incubation of the plates at 37°C, a single colony was picked and inoculated into a tube containing 4 mL of LB medium with zeocin (20 μg/mL). Cell cultures were grown overnight at 37°C with agitation (200 rpm). Afterwards cells were pelleted by centrifugation at 16,000 x g for 1 min. Supernatant was removed and the cells were resuspended in 2 mL DSM. Cell density was defined photometrically at 600 nm and a cell suspension with an optical density at 600 nm of 0.1 was inoculated into 100 mL Erlenmeyer flasks containing 30 mL prewarmed DSM and the antibiotic zeocin (20 μg/mL). After 48 hours of inoculation, samples of 200 μL from each culture were acquired for flow cytometry. Samples were centrifuged at 16,000 x g for 1 min with subsequent removal of the supernatant. Pelleted cells were resuspended in 500 μL 1 x PBS and stained with 2 x SYBR1 as mentioned above. The same process was repeated at 48, 56, 72, 80 and 96 hours. All experiments were conducted in three biological replicates.

### Flow cytometry and FACS

Cytometric analyses were done using a Sony LE-SH800SZBCPL with a 488 nm argon laser. Gains for photomultipliers for the channels SSC and FL-1 (525/50 nm) were set on 40.0%, 39.0% and 51.0% respectively with a FSC-threshold of 0.20% and a window extension of 50. The FSC diode was set on an amplification level of 16/16 and sample pressure was set so that events per second (eps) were kept under 30’000. For analysis and plotting, areas of scattering- and fluorescence signals were brought to a near-normal form by transforming over the inverse hyperbolic sine. For each analysis, 10⁵ events were evaluated. All analysis files together with the respective fcs files can be found on GitLab under https://gitlab.com/sporesort/.

For cell sorting, the same settings as for cytometric analyses were used, however eps were kept under 12’000. Agglomerates were excluded based on channels FSC-H/FSC-W as well as SSC-H/SSC-W.

### Data analysis and clustering

R version 3.5.1 was used running on Ubuntu 16.04.5 LTS. R package *flowCore_1*.*48*.*1* [37] was used to import fcs files and exclude agglomerates from analysis. Agglomerates were gated out by fitting bivariate normal distributions to channel pairs FSC-W/FSC-H and SSC-W/SSC-H using flowCores build-in function *norm2Filter* to detect outliers. For this, the default settings were used, so that the fast minimum covariance determinant (MCD) estimator [38] in the *covMCD* implementation from package rrCov_1.4–7 [39] with *scale-factor=1* was called. As one of the most common algorithms for outlier detection [40], MCD is most suitable for elliptical, symmetric, unimodal distributions. In this implementation, it is based on minimizing the determinant of the covariance matrix of a subset containing half of all data points in the dataset. Geometrically, this is equivalent to minimizing the volume of the ellipsoid represented by the covariance matrix given the subset of the data.

For the artificial 1:1 mixtures of spores and cells, exploratory visualization was done with *ggcyto_1*.*10*.*0* [41]. To evaluate the distribution of cells and spores in terms of their distances and spread, a mixture of two univariate normal distributions was assumed. Normal distributions were fitted using Mclust from the mclust_5.4.3 package [42] with *g=2* components (cells and spores). The model output included the two means and the two standard deviations for both the predicted distributions of cells and spores as well as the scaling parameters lambda. The quality of the staining was then evaluated by calculating the distance μ_HP-LP_(1) between the two distributions and pooling the standard deviation σ_HP-LP_ (2).

$$\mu_{HP-LP}=\mu_{HP}-\mu_{LP}$$ (1)

$$\sigma_{HP-LP}=\sqrt{\frac{\sigma_{HP}^{2}+\sigma_{LP}^{2}}{2}}$$ (2)

Subsequently, thresholds between the distributions for visualization were determined by deducting the higher signal population from the lower signal population and determining the root of the resulting function with *uniroot*.

Gaussian mixture models can be thought of as a prototype classification method, which employ a set of points in feature space (prototypes) to represent the dataset. The points in the dataset are then classified according to their proximity to the prototypes, each of which carries a unique class label. Proximity is most commonly measured as l2-norm, ie. the euclidean distance. For Gaussian mixture models, each prototype is the center of a gaussian density with an associated covariance matrix determining the variance and shape of the density [43].

Finally, GMM was used in the mclust implementation to identify clusters of spores, endospores and vegetative cells. The number of centers was set to g = 3 (corresponding to the three mentioned clusters) with all other settings being set to default. To identify clusters of spores, endospores and vegetative cells, GMM was applied on a reference dataset of a biological sample containing all three populations. The resulting model was then used to predict to assign cluster labels for all other samples. Visualization was subsequently done using *viridis_0*.*5*.*1* [44], *ggridges_0*.*5*.*1* [45], *cowplot_0*.*9*.*4* [45,46] and the *tidyverse_1*.*2*.*1* [47] containing *forcats_0*.*4*.*0* [48], *stringr_1*.*4*.*0* [49], *dplyr_0*.*8*.*0*.*1* [49,50], *purrr_0*.*3*.*2* [49–52], *readr_1*.*3*.*1* [49–51], *tidyr_0*.*8*.*3* [53], *tibble_2*.*1*.*1* [53,54] and *ggplot2_3*.*1*.*0* [53–57].

### Microscopy

For phase contrast microscopy 5 x 10⁶ events were sorted in 15 mL tubes coated with 5% BSA (Carl Roth, Germany). Sorted samples were transferred in 20 mL centrifugal concentrators of 30 kDa molecular cutoff and centrifuged at 3,400 x g for 5 min. Agarose pads were prepared on glass slides with 1% agarose standard (Carl Roth, Germany). Microscopy slides were prepared by pipetting 3 μL of the concentrated sample suspensions on top of the agarose pads placed on glass slides and covering with glass coverslips. Samples were observed with Axio Vert.A1 (Carl Zeiss) inverted microscope equipped with 100 x oil immersion objective and 10 x ocular magnification. Images were captured with AxioCam ICm1 (Carl Zeiss) camera using ZEN lite 2011 software (Carl Zeiss).

## Results

### Measurement of cells and spores from different samples

To develop a robust flow cytometric method for separation of *B*. *subtilis* vegetative cells and spores, separate samples of vegetative cells and purified spores were initially tested. Samples were respectively stained with SYBR1 and SYBR2 as described in materials and methods and subsequently analyzed by flow cytometry.

**Fig 1:**
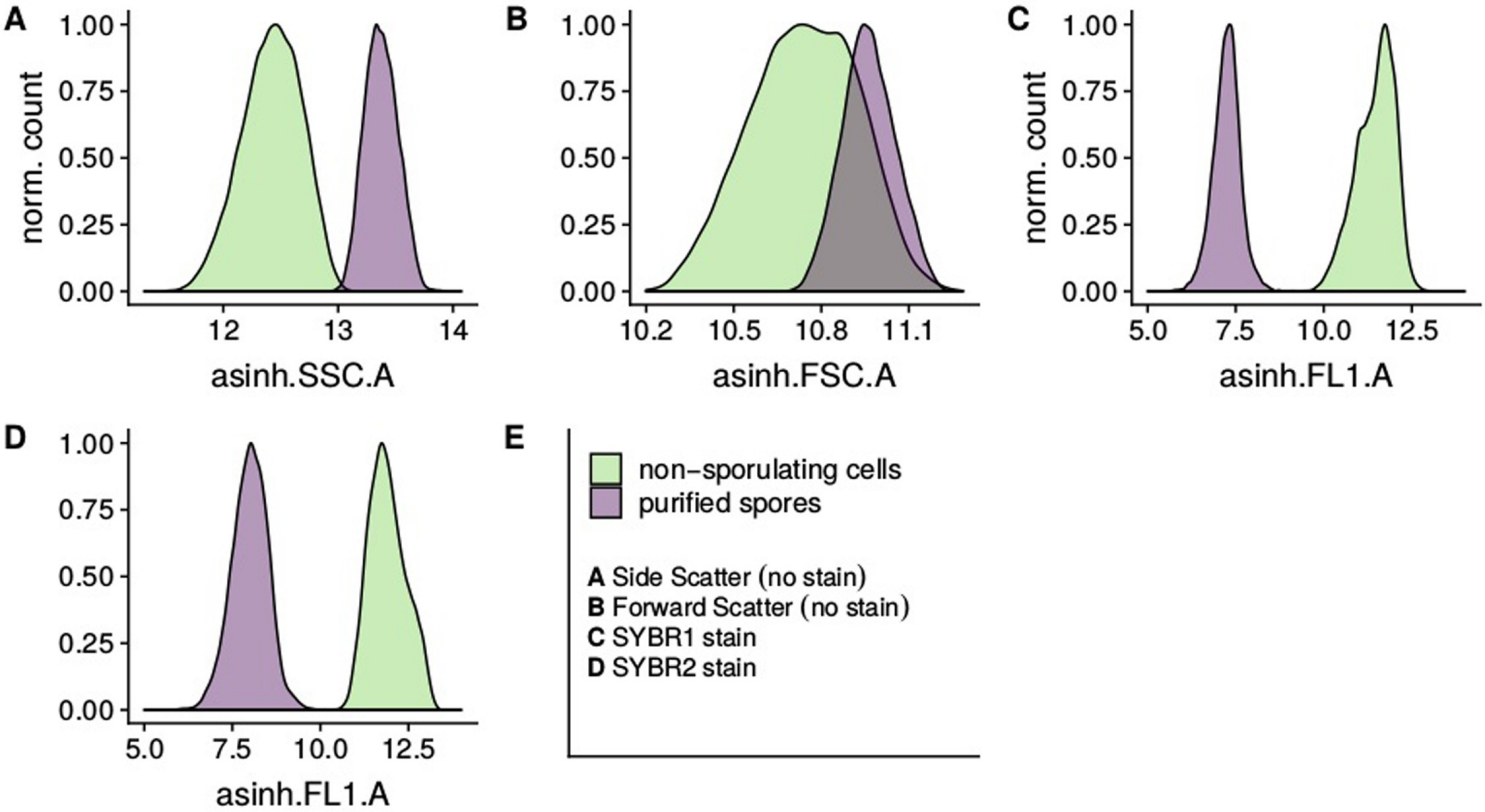
FI and scatter signals of *B*. *subtilis* vegetative cells and purified spores measured separately. To measure cells, the non-sporulating Bs02005 strain was employed. Spores were isolated from a culture of Bs02003 according to Nicholson and Setlow, 1990 [35] resulting in a yield of approx 90%.

As shown in Fig 1A and 1B, spores exhibit higher SSC and FSC than cells, however differences in scattering behavior alone do not provide clear distinction of the two populations. Staining of samples with fluorescent dyes yielded better separation on the respective channels (Fig 1C and 1D).

### Optimization of staining method

To assess the capacity of separating spores from cells coming from the same sample, a 1:1 mix of vegetative cells and purified spores was stained with different concentrations of dye. Subsequently, samples were measured after different incubation times and cell viability was quantified by sorting of cells and spores.

**Fig 2:**
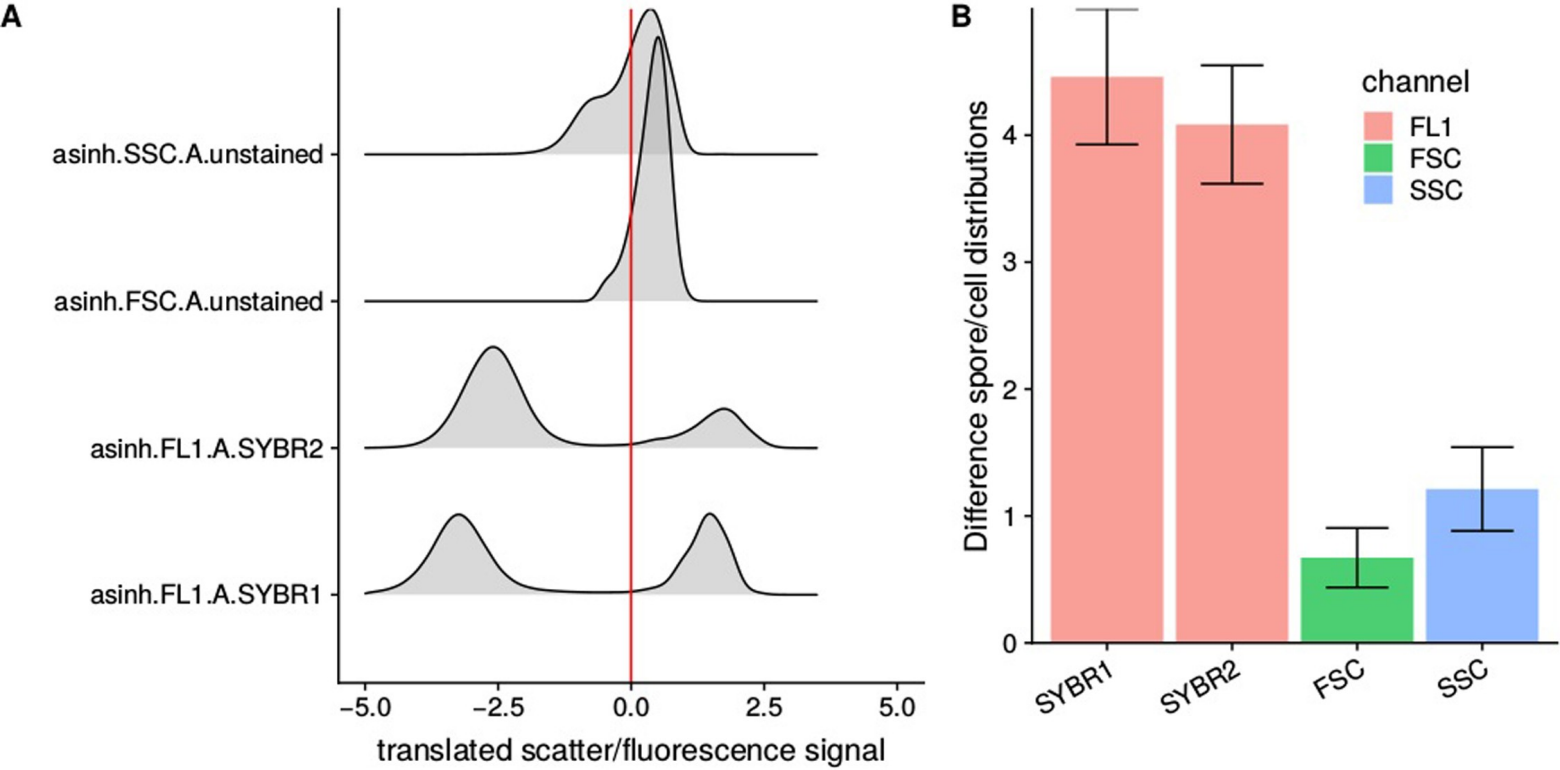
Comparison of different dyes and channels used for optimal separation of cells and spores (time = 30 minutes, concentration = 2 fold). (A) Raw data and cutoff values based on GMM. X-axis shows translated signal, so that the threshold is always at 0. (B) Distance between normal distributions as predicted by GMM in the respective channels with error bars showing the pooled standard deviation. The same metric was used to evaluate the effect of staining time, stain concentration (S1 Fig).

Details of the separation of putative spore and cell populations by Gaussian mixture modeling as described in material and methods section. The distance between the distributions of the two populations is shown in Fig 2 together with pooled standard deviation. Overall, staining with SYBR1 results in the best resolution between cells and spores. Treatment with SYBR2 resulted in smaller difference, whereas minimum difference was observed when FSC was used for separation, which is in line with previous experiments.

Variation of staining time as well as stain concentration yielded only minor differences (S1A Fig). Effects of the staining procedure on cell and spore viability was assessed by colony forming units as shown in S1B Fig. As a 2-fold concentration after 20 minutes of staining with SYBR1 appears to be sufficient for separation and does not result in significantly increased cell death, this concentration was employed for further investigations.

### Classification and cell sorting of sub-population

**Fig 3:**
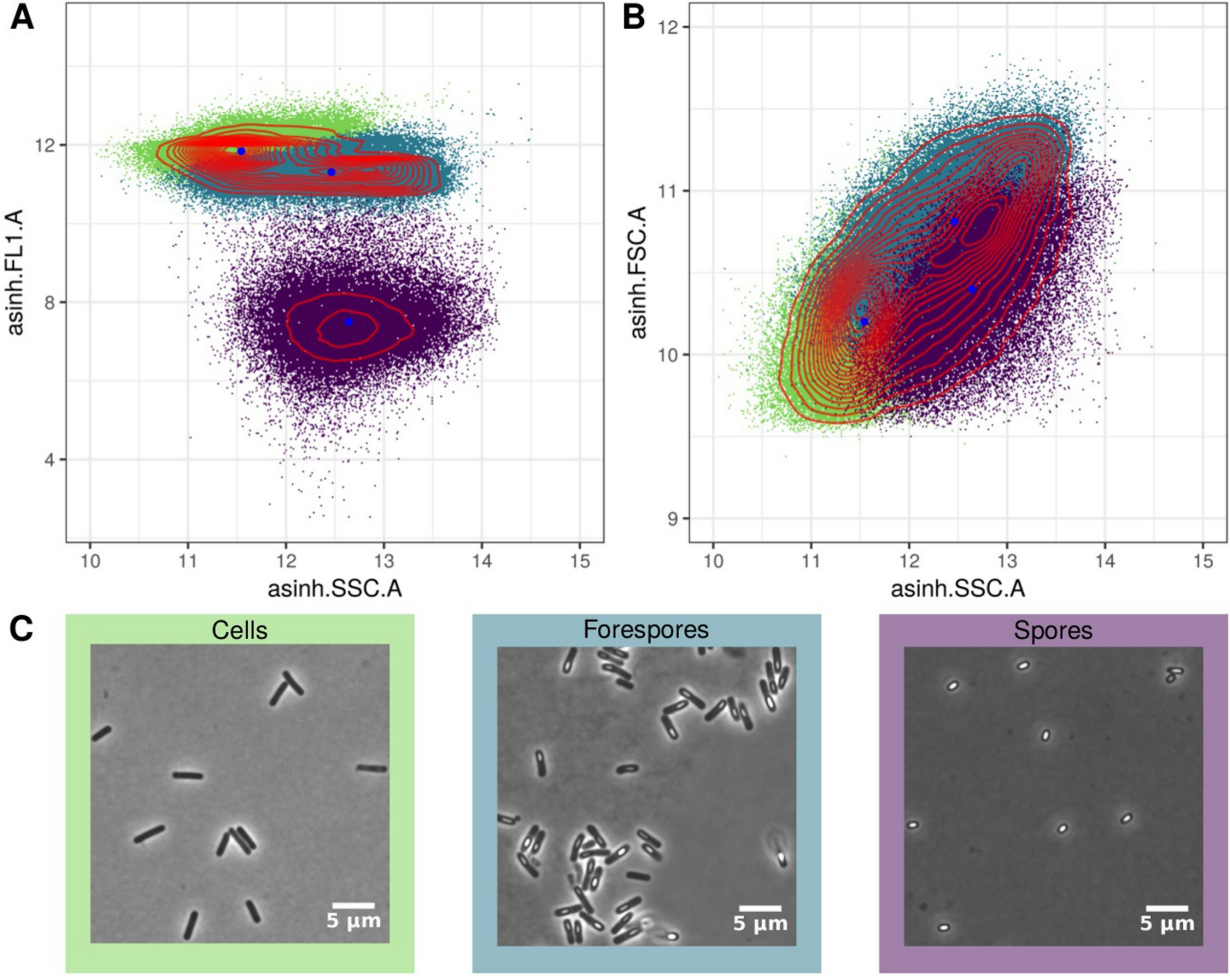
Scatter plots with color indicating the classification. Hyperbolic-sine transformed side scatter (SSC) and fluorescence in FL1-A channel are shown in (A), whereas (B) shows front- and side scatter (FSC, SSC). Color of the event indicates the respective cluster as predicted by GMM. Cluster centers are shown in blue and red lines show 2d kernel densities. (C) Corresponding microscopy images of the respective subpopulations are shown. Subpopulations were subsequently isolated by FACS.

To assess whether the developed method allowed for discrimination of an intermediate stage in the spore formation, sporulating cultures of Bs02003 were tested. Two distinct populations were observed, differing strongly in fluorescence, somewhat in SSC and little in FSC (Fig 3A and 3B). One of the two populations follows a bimodal distribution indicating the existence of two different sub-populations. The centers of the three clusters are shown in red. To validate that the three discovered subpopulations actually constitute the putative sporulation phases, 5 x 10⁶ events from each cluster were sorted and examined by phase-contrast microscopy (Fig 3C). Cells found in the respective samples matched the expected pattern of the subpopulation of cells, mother cells containing forespores and spores.

### Analysis of sporulation dynamics

The optimized method was applied for quantitative evaluation of sporulation dynamics of *B*. *subtilis* strains harboring different genome modifications. Initially the sporulation dynamics of strain Bs02018, utilized for display of sfGFP on the surface of spores, was investigated and compared to two other strains harboring similar genomic backgrounds (Bs02003, Bs02020). All three strains carry deletions of both *cwlD* and *sleB*, leading to a germination deficient phenotype [24–26]. Strain Bs02020 additionally harbors deletion of *cotA* which is responsible for the brownish pigmentation of spores [29].

**Fig 4:**
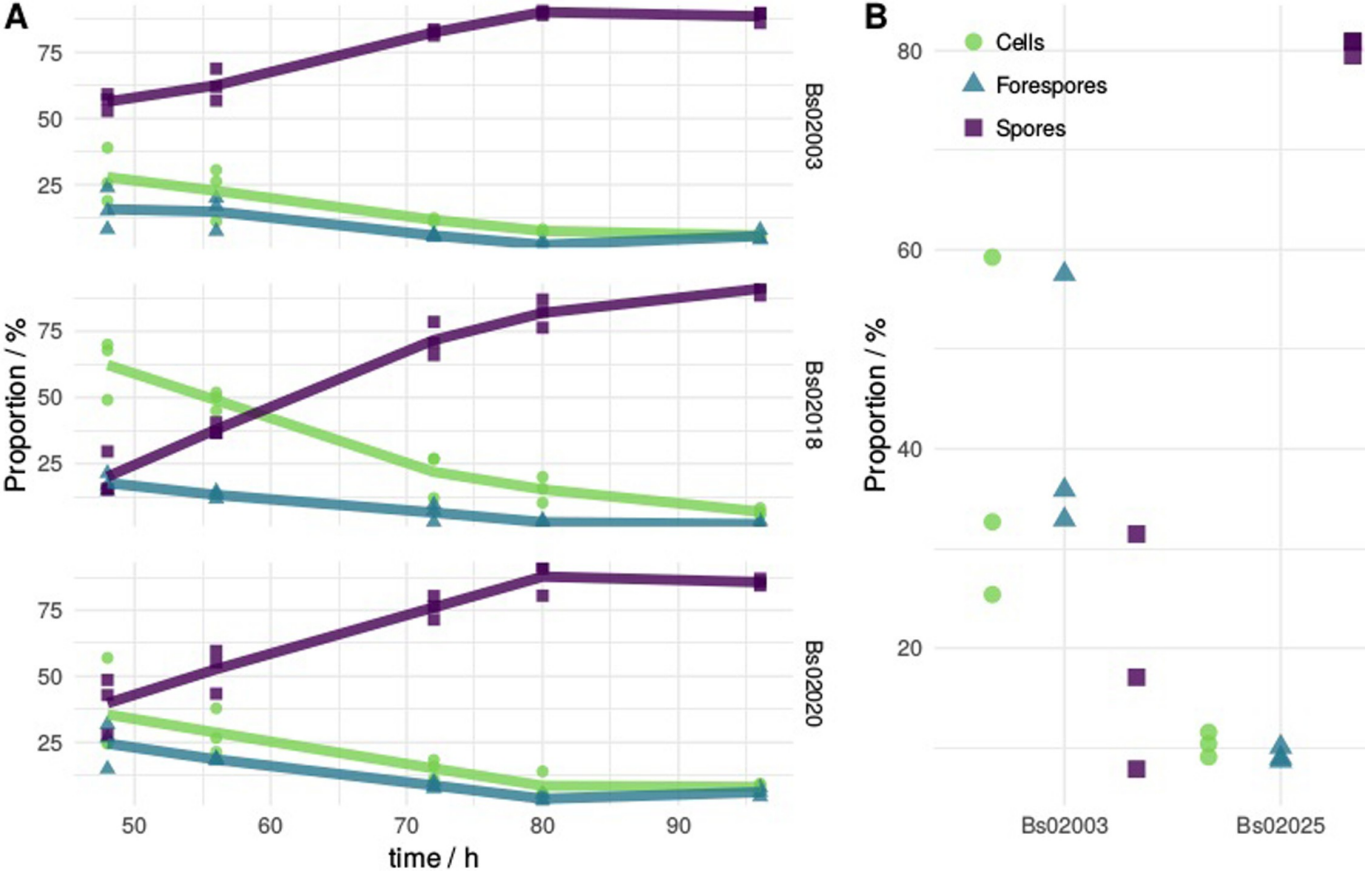
Analysis of sporulation dynamics of *B*. *subtilis* strains harboring different genome modifications. (A) Shift of culture subpopulations containing cells, endospores and spores over time. *S*trains Bs02003, Bs02018 and Bs02020 are double mutants for *cwlD* [24] and *sleB* [25] whereas strain Bs02018 additionally expresses sfGFP as a fusion protein utilized for spore surface display. Strain Bs02020 additionally harbors deletion of *cotA* [29]. (B) Comparison of culture subpopulations of two *B*. *subtilis* mutants after 24 hours of cultivation. Strain Bs02025 contains deletions of *cwlD* [24] and *sleB* [25] and additionally harbors deletions of *spo0E* [31] and *skfA* [30] whereas Bs02003 serves as control.

For monitoring sporulation of the three strains, population sizes were determined as described above for all strains and time points. In all cases, three distinct populations were observed as shown in Fig 4A. A clear difference in the sporulation pattern between strain displaying sfGFP (Bs02018) and the two other non-display strains is visible, with Bs02018 exhibiting lower sporulation efficiency.

As presented in Fig 4A, already after 48 hours, 59% of events in cultures of strain Bs02003 were spores as opposed to the other strains (35% and 44% respectively). In the course of 96 hours, maximum percentages of spores obtained for the three strains were 88%, 91% and 85% respectively. It is observed that for all strains, only a minority of events was made up by cells without endospores. Furthermore strains Bs02018 and Bs02020 exhibit lower sporulation efficiency in comparison to strain Bs02003. However, in earlier time points high variability was noticed, which made quantification more difficult.

The methodology was further used to evaluate the effect of *spo0E and skfA* deletions in strain Bs02025. Spo0E is a phosphatase which dephosphorylates the master regulator of sporulation, Spo0A~P and delays the process of sporulation [31] whereas spore killing factor SkfA, is part of the *skf* operon responsible for the production of toxin during sporulation [30]. Again, Bs02025 was compared to control strain Bs02003. The generated mutations are associated with higher sporulation efficiency (Fig 4B). After 24 hours of sporulation, cultures of Bs02025 already consisted of 80% spores while only 18% of events were classified as spores for the control strain.

## Discussion

In this work, we present a flow cytometric method for rapid quantification and isolation of subpopulations formed in sporulating cultures of *B*. *subtilis*. Spores consist of a thick peptidoglycan cortex, surrounded by a multilayered proteinaceous coat [58]. We hypothesize that the spore complex structure might be responsible for the increased SSC and FSC compared to the respective values for the vegetative cells. However scattering channels alone are commonly not sufficient for efficient resolution of the two populations. In contrast, Laflamme et al. were able to show separation and isolation of *Bacillus subtilis var niger* spores by employing UV induced autofluorescence [59] with a less common ultraviolet laser. However the necessity for special equipment, hampers the applicability of the described method.

Fluorescent staining has previously been used to investigate the sporulation dynamics of *Paenibacillus polymyxa* and clostridia respectively [60–62], as well for discrimination of vegetative cells from spores of *Bacillus licheniformis* contained in probiotic tablets [63]. In the present study, two different fluorescent dyes were tested for optimal separation of vegetative cells and spores. SYBR1 and SYBR2 are DNA binding dyes commonly used in microscopy and flow cytometry [64,65]. The data suggest that SYBR1 is the most suitable dye for separation of subpopulations.

Subjective gating and thresholding can lead to different results based on the operators subjective experience [66]. When subpopulations can be clearly distinguished, manual methods are mostly sufficient. However, if populations are poorly resolved, inter-lab reproducibility can be achieved better with automated gating [67]. Overall interest in automating these processes for flow cytometry has strongly increased over the past years. Thus, we employed different automated methods to allow for more reproducible and operator-independent separation of spore distributions.

In this work, we employed Gaussian mixture modeling (GMM) as a means to separate near-normal subpopulations of cells and spores separated on different channels. This allowed for a reduction in tested samples by measuring cell and spore phenotype in a single vial, which further guarantees the same staining environment for cells and spores as opposed to staining separately. GMM is being more routinely used for prediction of subpopulations [68,69], but is commonly restricted to cases, where signal distributions are at least near-normal. As scattering and fluorescence signals in flow cytometry often follow log-normal distributions, thresholding or clustering with this method can be a useful tool for separation. Further, Lee and Scott [70] showed, that even in the case of censored or restricted data, which frequently occurs in application if the limits of linear detector range is reached, GMM can be modified to suit these conditions.

By using GMM on a reference sample of spores, cells and mother cells containing forespores, the respective subpopulation of any other sample in the respective experiment set was determined by assigning each point to its closest center. In our case, there was little to no shift of populations over the respective independent variable (e.g. time, triplicate). Consequently, Euclidean distance to the calculated centers could be used to classify subpopulations. The three detected clusters were attributed to different phases of sporulation based on the following hypothesis:

Vegetative cells display high permeability for SYBR1 and exhibit associated high fluorescence intensity. During formation of the endospore, this permeability and FI remain similar, however the emerging protein structure of the coat might increases the granularity and hence the measured SSC. Also, an increase in average FSC might be attributed to a minor increase in average cell size caused by intracellular formation of the coat. Subsequently, transition from endospore formation to spore release facilitates reduction in FSC and correspondingly cell size, possibly due to smaller size of the spore in comparison to the cell/spore complex. As the spore formation is completed, permeability for SYBR1 decreases due to the presence of the coat or the cortex [71], leading to a drop in FI. Evidence for this hypothesis was gathered by confirming the identity of the respective subpopulations.

It has been demonstrated that the presented method can be utilized for at-line monitoring of sporulation. The optimized method was applied on sporulating cultures of different strains to assess the effect of genomic modifications to the dynamics of sporulation. Quantification of the three major populations formed during sporulation showed a reduction in sporulation efficiency of strain Bs02018, utilized for display of sfGFP on the surface of spores. Based on these results, we assume that expression of sfGFP poses an additional metabolic burden to the already energy consuming process of sporulation. We further demonstrated that a knockout of genes *skfA* and *spo0E* increased the sporulation efficiency. These findings are in line with the results reported for *spo0E* deletion [72].

In summary, flow cytometry combined with cell sorting allows for more rapid quantification and isolation of cell populations compared to conventional methods. Our approach facilitates reliable and rapid separation of spores while reducing subjective and laborious gating. We anticipate our method to contribute to the ongoing research in the field of sporulation by allowing for high-throughput analysis using automated incubation and cytometry in microtiter plates as well as for optimizing spore display by enabling rapid characterization of relevant mutants.

## Supporting information

S1 Table Oligos employed in the present study. (PDF)

S2 Table Plasmids employed in the present study. (PDF)

S1 Fig Optimization and evaluation of staining procedure. **(A)** S1A Fig shows variations in dye concentration and staining time. Differences of the distribution means as predicted by GMM with error bars showing pooled standard deviations are displayed. **(B)** S1B Fig shows cell survival after the same staining procedures. The percentage of viable cells is calculated as share of colonies based on 210 events, which were sorted on an agar plate.
(TIFF)

S2 Fig Separation of mixed cultures. For all measured samples, dashed lines show the predicted normal distributions of mostly bimodal cell/spore distributions as determined by Gaussian mixture modeling. (A) S2A Fig shows samples after 30 minutes. (B) S2B Fig shows samples after 90 minutes of staining.
(TIFF)

S3 Fig Raw scatter plots of Fig 4B. Raw scatter plots of data used in Fig 4B: triplicates of Bs02003 and Bs02025 shown as scatterplots of side scatter and SYBR1 fluorescence.
(TIFF)

S1 Text Viability assessment after staining. (PDF)

## Abbreviations

DSM: Difco sporulation medium
Eps: events per second
FACS: fluorescence activated cell sorting
FI: fluorescence intensity
FL1-A: fluorescence channel 1, signal area (525/50 bandpass) for SYBR1 and SYBR2
FSC-A: forward scatter, signal area
FTIR: Fourier-transform infrared spectroscopy
GMM: Gaussian mixture models
LB: Lysogeny broth
PBS: phosphate buffered saline
sfGFP: superfolder green fluorescent protein
SYBR1: SYBR Green 1
SYBR2: SYBR Green 2
SSC-A: side scatter, signal area
