# Supplementary material for: Quantification and isolation of Bacillus subtilis spores using cell sorting and automated gating
Source: PLoS One. 2019 Jul 29;14(7):e0219892. doi: 10.1371/journal.pone.0219892 (PMC6663000; doi:10.1371/journal.pone.0219892)
Supplement: S1 Table — (PDF) [file pone.0219892.s001.pdf]

S1 Table. Oligos employed in the present study

Oligo number	Oligo name	Sequence (5’-3’)
02024	*cwlD*_front_fw	AGATCTTCCGGATGGCTCGAGTTTTTCAGCAAGATCTTCATTTGGGCGAATTTCC
02025	*cwlD*_front_rv	TACGAACGGTAGGCCTCGAGGATCCAATTCGTGATTACCGCATGCGAAGCTTAC
02026	*cwlD*_back_fw	CTCTAGATGAATTGGTGAAGCGCTGATCAGAAAAAGGAGACCCTCCGGAGTAATG
02027	*cwlD*_back_rv	AGCTGAGAATATTGTAGGAGATCTTCTAGAAAGATCCGCATCAATTAAACCAACC
02012	*sleB*_front_fw	AGATCTTCCGGATGGCTCGAGTTTTTCAGCAAGATCTGCACACAAAAAAGCCGC
02023	*sleB*_front_rv	TACGAACGGTAGGCCTCGAGGATCCAATTCGTGATCTTCGGGGTTTTTGGAGG
02014	*sleB*_back_fw	CGGTAGGCCTCTAGATGAATTGGTGAAGCGCTGATCATTTCCCGGCTGAATTTGC
02022	*sleB*_back_rv	AGCTGAGAATATTGTAGGAGATCTTCTAGAAAGATGCAGAACCTATATATAAAGACC
02039	*spoIIGA*_front_fw	AGATCTTCCGGATGGCTCGAGTTTTTCAGCAAGATCCTATTGCTTCCTTCGCTTAG
02040	*spoIIGA*_front_rv	TACGAACGGTAGGCCTCGAGGATCCAATTCGTGATCGAGGAAGTATAATGAGAGGATATA

02089	*cotA*_frn_OH_fw	AGATCTTCCGGATGGCTCGAGTTTTTCAGCAAGATCCATGTTCATCTGTTCTTTTTGG
02090	*cotA*_frn_OH_rv	TACGAACGGTAGGCCTCGAGGATCCAATTCGTGATCCTCTAGCAGGCAAGTTTTTTC
02091	*cotA*_back_OH_fw	CGGTAGGCCTCTAGATGAATTGGTGAAGCGCTGATACAAAATCCTAAACGGCAGG
02092	*cotA*_back_OH_rv	AGCTGAGAATATTGTAGGAGATCTTCTAGAAAGATTTGCAGAAAGAAATGAAGCG
02158	*spo0E*_front_fw	AGATCTTCCGGATGGCTCGAGTTTTTCAGCAAGATCCACTTTTCATATTGTGACAGTTC
02159	*spo0E*_front_rv	TACGAACGGTAGGCCTCGAGGATCCAATTCGTGATGCTAAGAAATAGGAAACAAGTTTG
02160	*spo0E*_back_fw	CGGTAGGCCTCTAGATGAATTGGTGAAGCGCTGATATAAAGAGAGAGCTTTTCGGAAG
02161	*spo0E*_back_frv	AGCTGAGAATATTGTAGGAGATCTTCTAGAAAGATGCATTGCCATTCGATTTGC
02047	*cotB*_front_pJET_OH_fw	AGATCTTCCGGATGGCTCGAGTTTTTCAGCAAGATAGCTCAGTTTAAGTCAGAAGTG
02062	*cotB*_front_SSS_OH_rv	TACGAACGGTAGGCCTCGAGGATCCAATTCGTGATCGCGAATACCCATTTTCACG
02049	*cotB*_back_SSS_OH_fw	CGGTAGGCCTCTAGATGAATTGGTGAAGCGCTGATGGAGGAGCATCAGATA

		AAGTG
02050	*cotB*_back_pJET_OH_rv	AGCTGAGAATATTGTAG GAGATCTTCTAGAAAGA TGTATACGGTGTTTGTTC AGTTG
02124	*PcotYZ*_pksx_OH_fw	TTATAAAAATTGCCCTCT CATTTTTTTCTTGTTACC CAAGCATATGATGAATAT ATAGACG
02171	*PcotYZ*_cotB_rv	ATTTCATTATTTGAATGA TATTTCATTCTCCTCTTG CTCATTGATTTCAGCTCC TTCTTTATAGG
02173	*cotB*_OH_Pcoty_fw	AAGTCAAATACCCTATA AAGAAGGAGCTGAAAT CAATGAGCAAGAGGAG AATGAAATATC
02172	*cotB*_OH_linker_rv	ACTTCCACCACCTCCAC CACTTCCACCACCTCCA CCAAATTTACGTTTCCA GTGATAGTC
02066	sfGFP_linker_OH_fw	GGTGGAGGTGGTGGAA GTGGTGGAGGTGGTGG AAGTATGCGTAAAGGCG AAGAG
02067	sfGFP_lox71_OH_rv	AATAACTTCGTATAATGT ATGCTATACGAACGGTAT CATTTGTACAGTTCATCC ATACC
14089	Spec-mid-fw	CAATAGCCAAATCAGGA TCATAGC
14090	Spec-mid-rv	GGACAAATTCAGGAACC AAGC
14249	SSS-l71_fw	ATCACGAATTGGATCCT CGAG
14250	SSS-l66_rv	ATCAGCGCTTCACCAAT TCATC
23043	pJetFor	ATCTTGCTGAAAAACTC GAGC
23044	pJetRev	ATCTTTCTAGAAGATCTC C

11290	front_rv	CCGATAACTTCGTATAATGTATGCTATACGAACGGTAGGAGCTCACCCCTAATTTTCC
11291	front_fw	GAAAGGAGGAAGCGGAAGAATGAAGTAAGAGGGCGTAAACCTGAACATCCGCG
11403	back_fw	GGCATAACTTCGTATAGCATACATTATACGAACGGTAGGTTGAATATTATGATGAAACGGTCC
11404	back_rv	GGATTGGAGGAGTATGGCCACTAATACTAAGTTCAGCGTATCCATAACCTGCCCGATC
