# Supplementary material for: Quantification and isolation of Bacillus subtilis spores using cell sorting and automated gating
Source: PLoS One. 2019 Jul 29;14(7):e0219892. doi: 10.1371/journal.pone.0219892 (PMC6663000; doi:10.1371/journal.pone.0219892)
Supplement: S2 Table — (PDF) [file pone.0219892.s002.pdf]

S2 Table. Plasmids employed in the present study

Plasmid	Backbone	Integration site	Resistance	Reference
p02002	pJET	*cwlD*	spec	This study
p02004	pJET	*spoIIGA/sigE*	spec	This study
p02005	pJET	*sleB*	spec	This study
p02006	pJET	*cotB*	spec	This study
p02013	pJET	*cotA*	spec	This study
p02023	pJET	*spo0E*	spec	This study
p02030	pJK179	*pksX*	spec	This study
p11083.2	pJET	*skfA*	spec	PhD thesis of Silke Hackenschmidt
