# Supplementary figures and images for: Quantification and isolation of Bacillus subtilis spores using cell sorting and automated gating
Source: PLoS One. 2019 Jul 29;14(7):e0219892. doi: 10.1371/journal.pone.0219892 (PMC6663000; doi:10.1371/journal.pone.0219892)

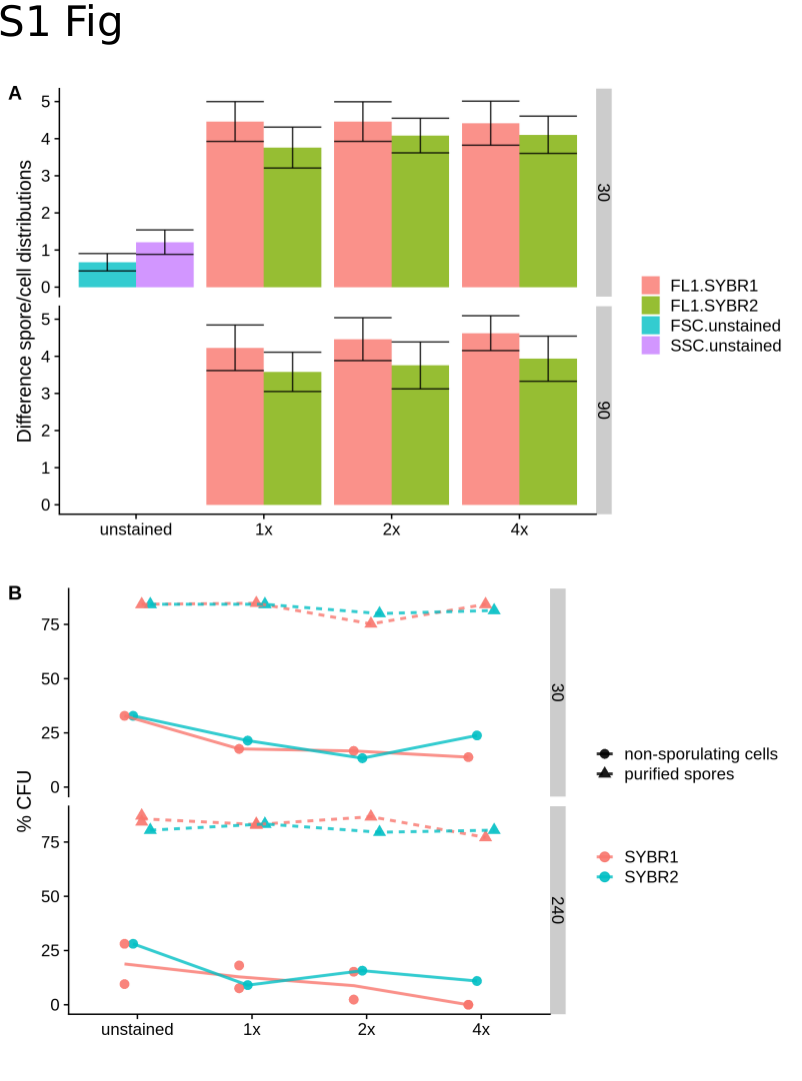

Supplement: S1 Fig — (A) S1A Fig shows variations in dye concentration and staining time. Differences of the distribution means as predicted by GMM with error bars showing pooled standard deviations are displayed. (B) S1B Fig shows cell survival after the same staining procedures. The percentage of viable cells is calculated as share of colonies based on 210 events, which were sorted on an agar plate. (TIFF) [file pone.0219892.s003.tiff]

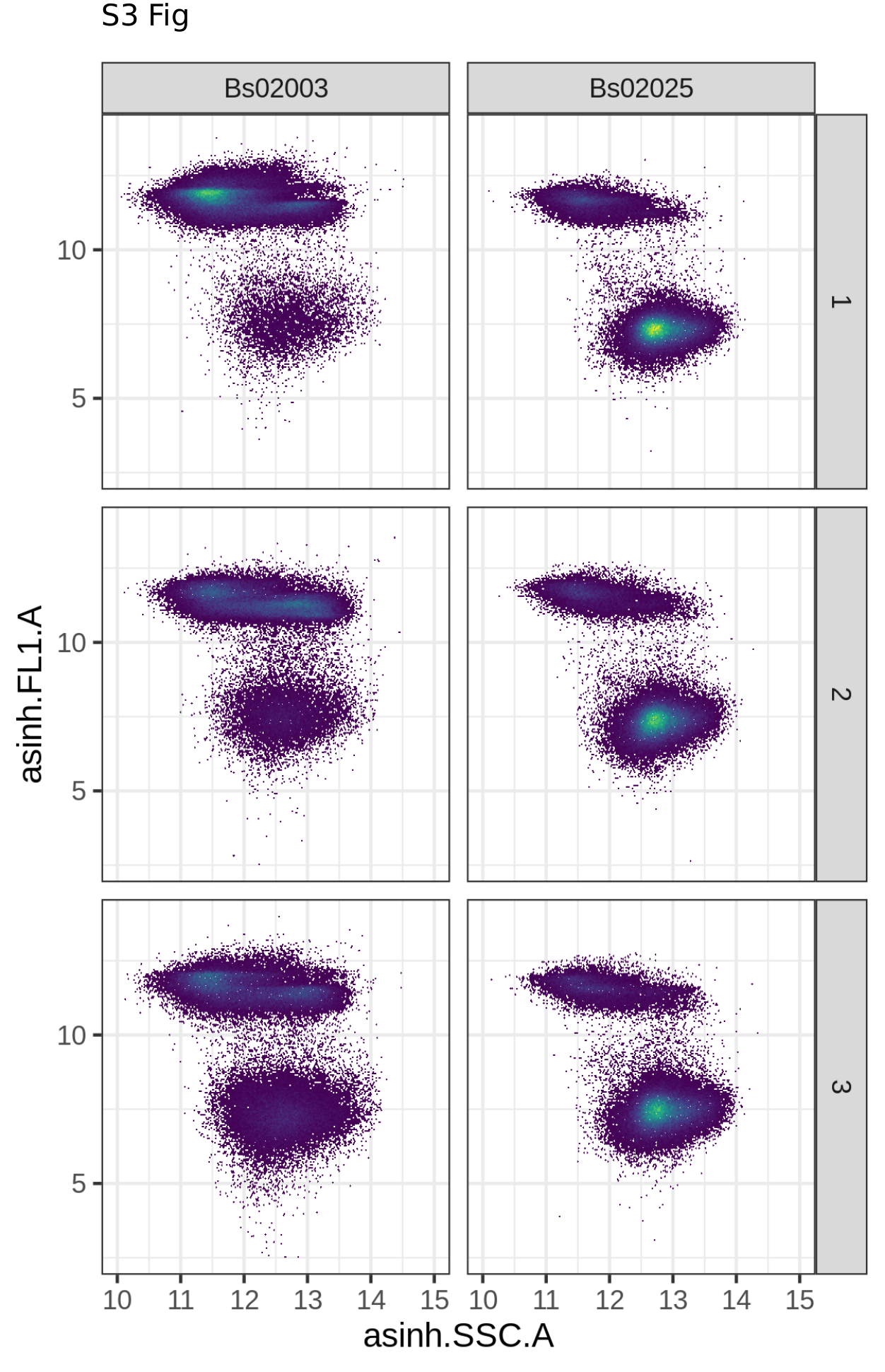

Supplement: S3 Fig — Raw scatter plots of data used in Fig 4B: triplicates of Bs02003 and Bs02025 shown as scatterplots of side scatter and SYBR1 fluorescence. (TIFF) [file pone.0219892.s005.tiff]
