# Supplementary material for: Quantification and isolation of Bacillus subtilis spores using cell sorting and automated gating
Source: PLoS One. 2019 Jul 29;14(7):e0219892. doi: 10.1371/journal.pone.0219892 (PMC6663000; doi:10.1371/journal.pone.0219892)
Supplement: S1 Text — (PDF) [file pone.0219892.s006.pdf]

S1 Text. Viability assessment after staining

To monitor the effect of staining in the viability of cells and spores, distinct samples of cells from sporulation deficient strain Bs02005 and spores from germinating strain Bs02002 were stained with SYBR1 and SYBR2 as mentioned in Materials and Methods section. Viability assessment was performed by sorting 210 events from each sample on an LB plate containing zeocin (20 mg/mL). Sorting was performed using a 100 µm microfluidics sorting chip for Sony SH800 (Sony), with the single cell three drops mode. Cells and spores were sorted on the plate according to the 384 well plate sort layout. Subsequently, plates were incubated overnight at 37°C. For analysis, the relative number of colonies in respect to the total number of sorted events was evaluated.
